# MERCs. The Novel Assistant to Neurotransmission?

**DOI:** 10.3389/fnins.2020.589319

**Published:** 2020-11-09

**Authors:** Olesya M. Shirokova, Pavel V. Pchelin, Irina V. Mukhina

**Affiliations:** ^1^Central Scientific Research Laboratory, Institute of Fundamental Medicine, Privolzhsky Research Medical University, Nizhny Novgorod, Russia; ^2^Department of Neurotechnology, Institute of Biology and Biomedicine, Lobachevsky State University of Nizhny Novgorod, Nizhny Novgorod, Russia

**Keywords:** synapse, endoplasmic reticulum, mitochondria-endoplasmic reticulum contacts, calcium homeostasis, neurodegenerative diseases, synaptic transmission

## Abstract

In neuroscience, much attention is paid to intercellular interactions, in particular, to synapses. However, many researchers do not pay due attention to the contribution of intracellular contacts to the work of intercellular interactions. Nevertheless, along with synapses, intracellular contacts also have complex organization and a tremendous number of regulatory elements. Mitochondria-endoplasmic reticulum contacts (MERCs) are a specific site of interaction between the two organelles; they provide a basis for a large number of cellular functions, such as calcium homeostasis, lipid metabolism, autophagy, and apoptosis. Despite the presence of these contacts in various parts of neurons and glial cells, it is yet not known whether they fulfill the same functions. There are still many unsolved questions about the work of these intracellular contacts, and one of the most important among them is if MERCs, with their broad implication into synaptic events, can be considered the assistant to neurotransmission?

## Introduction

Synaptic transmission is a complex process of communication between neuronal and target cells (Meriney and Fanselow, 2019). Many membrane structures and organelles are involved in synaptic transmission, including the synaptic plasma membrane and receptors, synaptic vesicles, structures in synaptic spines, mitochondria, endoplasmic reticulum (ER), and autophagosomes (Vos et al., 2010; Liang, 2019). Over the last decade it became evident that due to their typical elongated structure, mitochondria and the ER can form contacts with other organelles, thus modulating a broad spectrum of intracellular processes (Bravo-Sagua et al., 2014). Studies demonstrate the existence of a physical and functional connection between the ER and mitochondria via the junctions called mitochondria-endoplasmic contacts (MERCs) or, in case of an isolated fraction of these contacts, mitochondria-associated membranes (MAM) (Giorgi et al., 2015). MAMs are a product of the biochemical enrichment of mitochondria and ER membranes linked to each other and include many proteins and lipids that form MERCs (Janikiewicz et al., 2018). As well as in non-neuronal cells, MERCs were characterized in cells across the central nervous system, mostly in neurons (Gómez-Suaga et al., 2019), while, in astrocytes (Goebel et al., 2019), the data are scarce, and there is only indirect evidence of MERCs’ presence in microglia (Xie et al., 2017), and oligodendrocytes (Simpson et al., 1997). Due to their distribution and carried out functions, it is brought into question whether MERCs can play a significant role in synaptic transmission and serve as an important link connecting mechanisms in the pathogenesis of neurodegenerative diseases, thus serving as a promising therapeutic target. Still, among all membrane structures potentially involved in supporting neurotransmission, the complex of MERCs remains to be the least studied.

Consequently, the purpose of this review is to designate the possible role of MERCs in supporting active neurotransmission under normal conditions and to outline how altered MERCs function can contribute to the development of failing neurotransmission in neurodegenerative diseases. The question brought to the discussion is whether MERCs could be considered an important assistant to neurotransmission.

## MERCs Structure and Functions in Neuronal Cells

### Structure

Even though MERCs are shown to be present in multiple parts of the neuron (Lee et al., 2018), the precise characteristics of these contact sites remain to be under discussion.

In general, MERCs comprise a set of chemical bonds, many of which are found both in the ER and mitochondria. Other proteins are localized to the membrane of only one organelle, but still are concentrated precisely at the contact site. The huge variety of proteins (>1000), discovered during the fractionation (Poston et al., 2013) of MAM, can be explained by the imperfection of techniques that cannot isolate the proteins only in the area of interaction. However, it is considered that the main connecting link of MERCs is the tripartite complex, which transports ions of calcium (Ca^2+^) and is composed of inositol 1,4,5-triphosphate receptor (IP3R) on the ER membrane [see (Serysheva, 2014) for more details on the channel structure], voltage-dependent anion channel 1 (VDAC1) on the outer membrane of mitochondria and the connecting cytosolic chaperone of glucose-regulated protein 75 (GRP75; a member of the heat shock protein family, 70 kDa) (Honrath et al., 2017). Among other MERCs functional protein complexes are mitofusin homo- and heterodimers (Mfn1-Mfn2) (function: modulation of mitochondrial mobility, auto- and mitophagy) (de Brito and Scorrano, 2008; Filadi et al., 2015), the complex of ER protein VAPB (vesicle-associated membrane protein-associated protein B/C) and mitochondrial PTPIP51 (protein tyrosine phosphatase-interacting protein 51) [function: molecular scaffold between ER and mitochondria related to Ca^2+^-exchange (Liu and Zhu, 2017; Paillusson et al., 2017), autophagy (Gomez-Suaga et al., 2017)], the complex of ER chaperone protein BAP31 (B-cell receptor-associated protein 31) and mitochondrial fission protein FIS1 (Fission 1) (function: transmission of apoptotic signals from mitochondria to ER) are also distinguished among the functional complexes of MERCs (Iwasawa et al., 2011; Krols et al., 2016), as well as many other proteins (Raturi and Simmen, 2013).

Cleft width is one of the key characteristics that allow assessing the status and functional role of MERCs. According to the hypothesis, MERCs with 10 nm cleft width perform lipid transfer between organelles, ≈20 nm cleft width change their function to Ca^2+^ exchange. Nevertheless, there are also “inactive” MERCs ready for activation as soon as two membranes get close enough to each other. Similarly, MERCs with the 30 nm cleft can be either Ca^2+^-MERCs or inactive phagocytic MERCs. Thus, the dynamics of the contact structure will play a key role in regulating MERCs activity, despite the complex distribution of various types of mitochondria-ER contacts. At present, it is not yet clear, whether there is a single mechanism that regulates the dynamics of all mitochondria-ER contacts, or if MERCs have different organization initially (Giacomello and Pellegrini, 2016).

Another important structural characteristic of MERCs is their length. Despite the fact that the MAM proteome is rather conservative in various cell types (Wang X. et al., 2018), the length of mitochondria-ER contacts may differ depending on the specific cell type (Giacomello and Pellegrini, 2016). For example, in mouse fibroblast cell cultures, 2.25% of the mitochondrial membrane is in close contact (<20 nm) with the ER (Cosson et al., 2012), in HeLa cells – 5–20% (Rizzuto et al., 1998), about 7% in neuronal cultures (Martino Adami et al., 2019), and about 10% in lymphoma cells (Csordás et al., 2006). Researchers observed the ER completely covering the mitochondrion around the area of its division (Friedman et al., 2011). Even though the molecular pathways implicated in the change of MERCs length are not yet elucidated, this property of MERCs changes in pathological conditions (Leal et al., 2016). For instance, under conditions of mutant α-synuclein expression, the decrease in MERCs length leads to impaired Ca^2+^ homeostasis (Paillusson et al., 2017). The increased contact surface can lead to significant metabolic alterations (Bray, 2018). Such an increase was shown to elevate mitochondrial Ca^2+^ content and ATP production promoting the recovery of damaged axons (Lee et al., 2019).

### Functions

**FIGURE 1:**
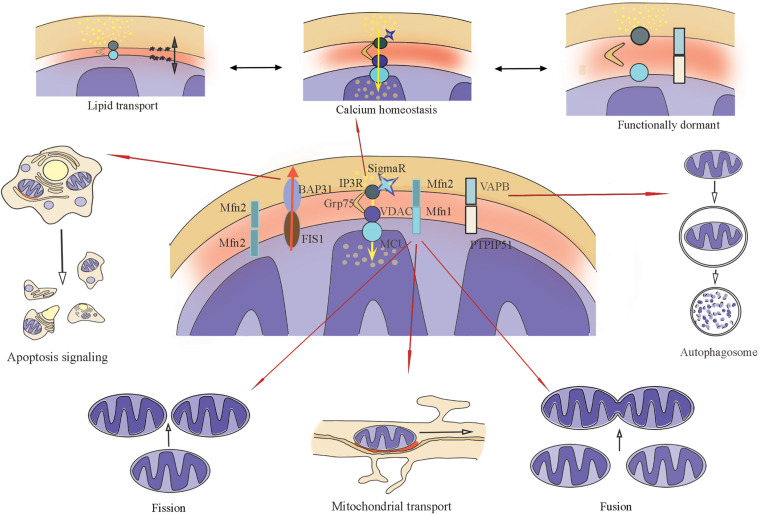
Main biochemical connections of MERCs (Mitochondria–endoplasmic reticulum contacts), their functions. ER-IP3R interacts with the mitochondrial channel VDAC1 together with chaperone GRP75, the main function is calcium transport. ER-VAPB interacts with mitochondrial PTPIP51 to form the molecular scaffold between ER and mitochondria related to Ca^2+^-exchange. ER-resident protein BAP31 interacts with mitochondrial protein FIS1. Together with PACS-2, this complex regulates the initiations of apoptosis. Mitofusin bridges (formed with Mfn1 and Mfn2) can be homodimers and heterodimers that change mitochondrial dynamics: mobility, fusion, and fission.

During active synaptic transmission, it is crucial to supply the synapse with a high enough level of energy (Harris et al., 2012), support the local Ca^2+^ balance, and provide the lipid synthesis to ensure the membrane fluidity, the renewal and modification of synaptic structures, such as vesicles, channels, and dendritic spines (Rodríguez-Berdini and Caputto, 2019). Since the main functions of MERCs include the influence on mitochondrial mobility and morphology (Tagaya and Arasaki, 2017; Giacomello et al., 2020), the exchange of calcium (Marchi et al., 2017; Müller et al., 2018; Cherubini et al., 2020) and lipids (Vance, 2014; Hanada, 2017) between organelles, and participation in processes of autophagy (Hamasaki et al., 2013; Garofalo et al., 2016; Tagaya and Arasaki, 2017), and apoptosis (Liu et al., 2014; Perkins and Ellisman, 2016; Figure 1), these intracellular contacts, localized in neurons and astrocytes, appear to be an indispensable assistant to neurotransmission.

#### Mitochondrial Fission and Mobility

Mitochondrial transport and distribution are especially crucial for neurons. Their specific structure with extended axons and dendrites requires high energy consumption at significant distances from the cell’s body, in particular, in synapses (Palikaras and Tavernarakis, 2020). For neurons to perform functions such as integration and signal transmission, the presence of a sufficient number of functionally active mitochondria is necessary (Sheng, 2017).

Due to the presence of mitofusins (Mfn1, Mfn2) (Bernard-Marissal et al., 2015) MERCs are involved in such physiological processes as mitochondrial fission and mobility inside the cell, auto- and mitophagy. However, the exact mechanism of mitofusins’ influence on the MERCs functioning and structure remains to be not fully understood (Filadi et al., 2015), and was questioned by many authors (Cosson et al., 2012; Filadi et al., 2015; Wang et al., 2015). Mfn−2 knockdown increases the length of ER-mitochondria contacts without affecting their number (Leal et al., 2016). Recently, a direct correlation between the form of mitochondria and the number/length of MERCs was found (Nagashima et al., 2019), and it was speculated that this intracellular interaction is necessary to maintain the complex shape of mitochondria.

#### Calcium Homeostasis

It is widely known that Ca^2+^ is considered to be an indispensable participant in mechanisms maintaining synaptic plasticity (Mateos-Aparicio and Rodríguez-Moreno, 2020). In cells, ER-mitochondria Ca^2+^ exchange is performed with MERCs GRP75 protein, which promotes the opening of IP3R calcium channels in the ER in the direct proximity to VDAC on the outer mitochondrial membrane (Honrath et al., 2017). The maintenance of this function was also shown to be supported by the VAPB-PTPIP51 complex (Paillusson et al., 2017) and PDZD8 protein (Hirabayashi et al., 2017).

Disruption of calcium transmission and other stress factors subsequently lead to the accumulation of proteins with impaired structure, which triggers unfolded protein response (UPR), the process mainly aimed to suppress the translation, to induce misfolded protein degradation, and to stimulate the formation of folding-related chaperones. However, if the cause cannot be eliminated, the UPR will initiate apoptosis. This is proved in numerous experiments on the depletion of such MAM proteins as Mfn2, SIGMA1R, and PACS-2 (phosphofurin acidic cluster sorting protein 2), as well as by the expression of the mutated form of VAPB, all of which induced UPR (Simmen et al., 2005, 2010; Langou et al., 2010; Muñoz et al., 2013; Prause et al., 2013; Bernard-Marissal et al., 2015).

#### Lipid Homeostasis

Lipids constitute an integral part of synaptic transmission, providing the formation of new membrane structures such as synaptic vesicles (Sudhof, 2004), modifying the function of ion channels (Carta et al., 2014), and the signal propagation along axons (Korinek et al., 2020). The functions of biochemical bonds formed by MERCs include phospholipid remodeling, cholesterol biosynthesis, phosphatidylserine synthesis. The intracellular conversion of free cholesterol creates a dynamic balance between membrane-bound and cytoplasmic cholesterol. Even though the molecular mechanisms that regulate the dynamics of the association between mitochondria and the endoplasmic reticulum remain as of yet unknown (Nagashima et al., 2019), researchers (Fujimoto et al., 2012) found an increase in the membrane association of these organelles, using methyl-β-cyclodextrin (MβC) (removes cholesterol from membranes). Therefore, under normal conditions cholesterol fulfills a restrictive role in the structural organization of MERCs (Fujimoto et al., 2012).

#### Synaptic Autophagy

Autophagy is known to regulate neurotransmission in the presynapse by the elimination of defective vesicles, and in the postsynapse by the disruption of structural proteins (Lieberman and Sulzer, 2020). Even though the data are scarce to date, it appears that MERCs are linked to autophagy during synaptic transmission. Firstly, membrane lipids, which are synthesized and modified in MAM, are known to be required for the formation of autophagosomes (Nagashima et al., 2019). Secondly, a high level of autophagy is required during neuronal stimulation for synaptic plasticity (Glatigny et al., 2019). Though it is worth mentioning that different forms of autophagy may be present in a cell’s body and synapses (Vijayan and Verstreken, 2017). The precise mechanisms of MERCs’ proteins involvement in synaptic autophagy are yet not known, however, the loss of MERCs and the disruption of autophagosome formation were observed when the expression of PACS-2 or Mfn-2 was reduced. On the contrary, VAPB and PTPIP51 knockdown also led to the MERCs loss, but in this case, autophagy was stimulated (Hamasaki et al., 2013; Gomez-Suaga et al., 2017).

Still, it is yet to be elucidated, whether functional and morphological types of MERCs have different biochemical bonds and interactions. It is quite likely that mitochondrial contacts with a rough or smooth ER can form individual subdomains with unique specialized functions, and only a part of these functions is currently studied using standard sample preparation method.

## MERCs in Synaptic Transmission

**FIGURE 2:**
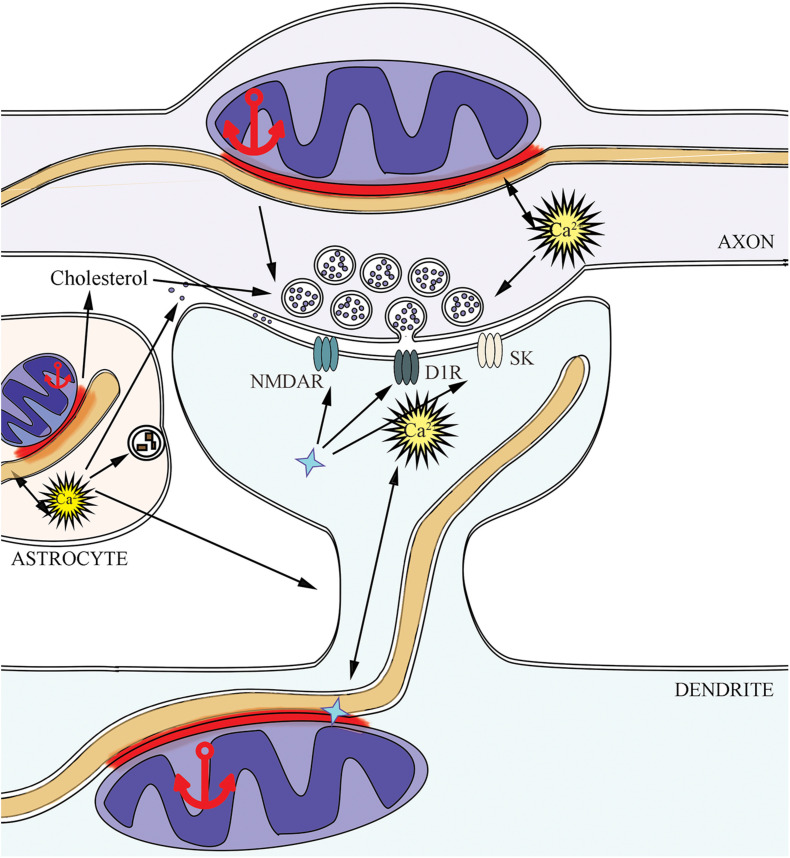
MERCs support synaptic activity in three sides: presynapse, postsynapse, and astrocyte. The implication of MERCs into supporting the active neurotransmission is connected with changes in calcium and lipid homeostasis, mitochondrial dynamics in pre- and postsynapse, due to the effects of SIGMA1R (shown as a blue star symbol) on ionic currents and membrane receptors. Dopamine D1 receptors – D1R, small conductance Ca^2+^-activated potassium channels – SK, NMDA-receptors – NMDAR.

Synaptic contact, or synapse, represents an interaction between two neurons in the central nervous system. Nevertheless, according to the modern concept, the synapse consists of three main parts: presynaptic terminal, postsynaptic terminal, and astrocyte process near the synaptic cleft (Perea et al., 2009). MERCs are present in pre- and postsynaptic parts (Gómez-Suaga et al., 2019), modulation of this connection in astrocytes changes their activity (Stephen et al., 2014), hence MERCs appear to affect the whole synapse as well (Figure 2). Individual synapses have different properties, physiology and morphology, and mitochondrial properties in synapses can vary significantly even between neurons within the same path (Hollenbeck, 2005). Further, we consider the role of MERCs in synapses, where mitochondria are present in the pre- or postsynaptic parts. The characteristics of mitochondria-free synapses, which can carry out neurotransmission using glycolysis as the energy source (Jang et al., 2016; Ashrafi et al., 2017), are not discussed in this review.

Most excitatory axons contact with dendritic spines, whereas inhibitory axons establish contacts mainly directly with dendrites. As is known, mitochondria are exceedingly rarely able to get into dendritic spines (Kasthuri et al., 2015). Therefore, it is most likely that MERCs do not directly influence all types of synaptic contacts, or rather not influence equally. The data of ultrastructural analysis confirm the presence of contacts between the ER and mitochondria in different parts of neurons (Wu et al., 2017; Hirabayashi et al., 2018) and astrocytes (Proulx and Borgmann, 2020). It is known that mitochondria in various parts of neurons are of different populations: in particular, in cell bodies and dendrites, these are complex mitochondrial networks, while in axons discrete mitochondria are present (Popov et al., 2005). Consequently, the functional role of MERCs in different parts of neurons can vary significantly.

At present, no in-depth study on the localization of MERCs and their characteristics within individual types of brain cells or in various parts of the central nervous system was carried out. Different types of tissues, their specific metabolic state are characterized by a certain amount of intracellular contacts. Moreover, the number of MERCs changes during the process of neurodegeneration (Leal et al., 2016) and aging (Calvo-Rodríguez et al., 2016).

### Presynaptic Support

Mitochondria in the axon exist in the form of two populations: mobile and stable. The high proximity of mitochondria to synapses is necessary due to the high energy demands during neurotransmission, therefore, there are mechanisms for mitochondrial retention near active synapses (Mironov and Symonchuk, 2006). Thus, it is known that mitochondria during the functional work of synapses are the main sources of energy (Harris et al., 2012). Different studies demonstrate various numbers of axonal mitochondria near the synapse: 36% (Chang et al., 2006) or 50% of axonal buds may contain mitochondria (Lewis et al., 2016), since mitochondrial mobility is regulated by mechanisms of the structural and functional plasticity in axons and synapses (Cai et al., 2011). Notably, one of the components of MERCs is Drp1 (Friedman et al., 2011), which indirectly influences neurotransmission. Drp1 is involved in mitochondrial fission (Friedman et al., 2011), contributes to the formation of synaptic vesicles pool in developing synapses (Li et al., 2008), participates in maintaining the bioenergetics of astroglial cells (Motori et al., 2013). As is known, presynaptic mitochondria, which number is related to Drp1 activity, regulate a short-term increase in the release of neurotransmitters during a period of frequent synaptic activity (Jonas, 2006) and regulate vesicle replenishment (Verstreken et al., 2005). Drp1 mutations lead to impaired mitochondrial dynamics and, as a result, to a lack of energy for synaptic neurotransmission during prolonged stimulation. Besides, the mobility of mitochondria affects local protein synthesis and hence the remodeling of synapses in general. However, taking into account the exceptional role of mitochondria in synaptic transmission, to date, the reason for the absence of mitochondria in some synapses remains to be unclear (Devine and Kittler, 2018).

The endoplasmic reticulum is present along the axon (Wu et al., 2017), allowing intracellular transport (Droz et al., 1975), and the regulation of calcium kinetics (Luarte et al., 2018). The role of MERCs is crucial for the regulation of calcium at the local subcellular level (Krols et al., 2016). A recent study (de Juan-Sanz et al., 2017) discussed the possibility of feedback between neuronal activity and the energy status of a whole cell. The authors made several remarkable conclusions. Firstly, the activity of neurons leads to the influx of Ca^2+^ to the axonal ER, not to the efflux. Secondly, axonal ER-calcium does not affect the cytosolic levels of Ca^2+^ in proximity to the synapse, except when under conditions of prolonged synaptic activity. Finally, a decrease in the axonal ER-calcium content leads to the activation of a temperature-dependent mechanism that modulates the release of the neurotransmitter, and this process is completely dependent on STIM (calcium sensor – Stromal interaction molecule), the integral protein of the ER and plasma membranes. Since one of the functions of MERCs is Ca^2+^ transfer from the ER to mitochondria through the tripartite protein complex, it appears to be possible to control the release of a neurotransmitter by targeting this intracellular contact (Yang et al., 2010).

A recent study highlights the role of membrane cholesterol in synaptic transmission; in particular, in action potential propagation along the axon, in the modulation of spontaneous release of glutamate at the presynapse, and the regulation of the opening and localization of NMDA receptors at the postsynapse (Korinek et al., 2020). It was shown before that cholesterol increases the number of vesicles present in the presynaptic terminal and the probability of their release, which leads to the general stimulation of presynaptic function (Dason et al., 2014). It also appears evident that the transfer of cholesterol across MAM from the ER to the mitochondrial membrane is necessary for steroidogenesis, correct organization of mtDNA, and to provide the intramitochondrial antioxidant protection (Arenas et al., 2017). The overall decrease in cholesterol leads to its reduced amount in MAM, which alters autophagy, as well as membrane fluidity and, as a result, a change in the ratio of amyloidogenic/non-amyloidogenic APP processing (Arenas et al., 2017).

### Postsynaptic Support

In the postsynaptic part, at the level of dendrites or dendritic spines, the effect of MERCs on synaptic transmission can be modulated in different ways. In axo-dendritic synapses, MERCs are close to the terminals. In dendrites, the important role of ER-mitochondria tethering protein PDZD8 was demonstrated in supporting the local Ca^2+^ homeostasis (Hirabayashi et al., 2017). On the contrary, a different situation is observed in axo-spine synapses, since mitochondria extremely rarely enter the dendritic spines (von Bohlen Und Halbach, 2009), but at the same time, the ER is found in many spines (Toresson and Grant, 2005).

It was demonstrated that such conservative MERCs proteins as PSS1 (phosphatidylserine synthase-1), PACS-2, IP3R3, SIGMA1R, Mfn2, and VDAC1 are ubiquitously localized to these contact sites in the cell bodies, processes, and synapses of neurons (Hedskog et al., 2013). MERCs are involved in supporting the active neurotransmission with SIGMA1R protein. The decrease in the Ca^2+^ concentration in the ER causes the dissociation of SIGMA1R from BiP (binding immunoglobulin protein), allowing the chaperone activity of the receptor to be activated. Further, SIGMA1R stabilizes IP3R3 to carry out Ca^2+^ exchange between organelles, supporting the bioenergetics of the cell (Hayashi et al., 2009). The IP3R-VDAC complex, stabilized by SIGMA1R, is of particular importance, since it is controlled from both organelles, notifying about its condition (Goetz et al., 2007). According to the modern concept (Kourrich et al., 2012), in the process of neurological disorders development, SIGMA1R is translocated from MERCs to other parts of the cell, as a result of which SIGMA1R binds to various ion channels, receptors, or kinases (NMDA, Src, D1R, PLC, DA transporter) and modulates their activity. SIGMA1R translocation triggers many reactions of this receptor with various ion channels (Schmidt and Kruse, 2019). SIGMA1R-NMDAR interactions are related to learning and memory. The effect on the function of NMDAR occurs due to the direct modulation of small-conductance Ca^2+^-activated potassium channels (Kourrich et al., 2012). SIGMA1R is also capable of suppressing the functions of NMDAR, for example, in retinal neurons, presumably via the PLC-PKC pathway, which depends on the Ca^2+^ concentration (Zhang et al., 2011). Free SIGMA1R exerts a significant influence on synaptic plasticity and the process of neurodegeneration. SIGMA1R is shown to stimulate BDNF-induced activation of phospholipase C (PLC)-gamma and the release of glutamate (Gómez-Suaga et al., 2018).

### Astrocytic Support

The third part of a synapse is the membrane of the glial process, although not all synapses are associated with glial presence as their functional part, for instance, in the neocortex there is only 50% of such synaptic contacts (Kasthuri et al., 2015). Three-dimensional reconstruction proves the existence of mitochondria-endoplasmic contacts in the processes of astrocytes (Goebel et al., 2019). However, when determining the role in modulating the synaptic transmission of MERCs located in the glial processes, not only should the percentage of synapses with glial processes in their composition be taken into account but also the cellular, molecular and functional diversity of the astrocytes themselves in the adult brain (John Lin et al., 2017). It remains not completely understood how intracellular contacts differ in these subpopulations of astrocytes. Besides, the activity of the synapses themselves also affects the morphology of the astrocytic processes (Morel et al., 2014). The importance of the connection between MAM-associated proteins and glia during synaptic transmission is proved by SIGMA1R or PACS-2 knockdown using siRNA in neuron-glial networks *in vitro*, which caused degeneration of processes in both neurons and astrocytes (Hedskog et al., 2013).

Mitochondria-endoplasmic reticulum contacts in astrocytes regulate mitochondrial mobility (Filadi et al., 2015). Mitochondria in astrocytic processes are mobile (Lovatt et al., 2007; Lavialle et al., 2011) and linger in places of high synapse activity, since there are a large number of glutamate transporters (Jackson et al., 2014). Moreover, the mobility of mitochondria is mediated by microtubules, but is mainly regulated by actin filaments with a change in the concentration of cytosolic calcium (Kremneva et al., 2013).

As is known, several astrocytic functions are triggered in dependence on certain concentrations of cytosolic Ca^2+^ (Bernardinelli et al., 2014). Among these functions are the release of gliotransmitters (Papouin et al., 2017; Sherwood et al., 2017), rapid removal of neurotransmitters from the synaptic cleft (e.g., glutamate, which causes excitotoxicity in high concentrations), mitophagy of neuronal defective mitochondria, elimination of synapses (Allen and Eroglu, 2017). According to the recent data, astrocytes are capable of transferring new mitochondria to axons (Hayakawa et al., 2016). Also, astrocytes are thought to be the most effective cholesterol synthesis sites in the brain (Nieweg et al., 2009). Astrocytes affect the progenitor cells of oligodendrocytes, and, accordingly, their viability (Varela-Echevarría et al., 2017). Since all of the mentioned functions are directly or indirectly influenced by a certain Ca^2+^ content in the cytosol, the role of MERCs, as a site of Ca^2+^ transfer between organelles, is highly significant. Thus, an increase in the level of GRP78 (BiP) protein, which is involved in calcium transmission between organelles, was used to protect mitochondria and contributed to the survival of astrocytes in acute ischemia and other pathological conditions, which include mitochondrial and ER dysfunctions (Ouyang et al., 2011).

As alluded to above, one of the MERCs main functions is the maintenance of lipid homeostasis (Vance, 2014). In mice with reduced lipid synthesis, especially in astrocytes, there are also a reduced release of cholesterol from astrocytes and impaired synaptic development with fewer amounts of presynaptic vesicles and defects in synaptic plasticity (van Deijk et al., 2017). MAM-associated enzymes provide the synthesis and transfer of phospholipids, cholesterol, and ceramides. With cholesterol depletion, the connection between two organelles increases (Fujimoto et al., 2012). However, at present, the exact mechanism by which cholesterol is transferred from the ER to mitochondrial outer membranes was not determined, and the role of MAM in this transport process requires further study, in particular, in astrocytes (Vance, 2014).

Reactive astrocyte activity following injury leads to the formation of glial scars, which prevent axonal growth, forming a physical and biochemical barrier (Chen et al., 2002; Varela-Echevarría et al., 2017). However, overexpression of mitofusins (one of the MAM components) triggers apoptosis in astrocytes (Liu et al., 2014) and other cell types (Wang W. et al., 2018). Thus, MERCs can be considered to be a potential therapeutic target to protect the nervous system from astrogliosis, which disrupts the regeneration of processes and interferes with synaptic transmission (Liu et al., 2018). Overexpression of Mfn2 changes the Bcl-2/Bax ratio (Wang W. et al., 2018). Bcl-2 regulates IP3R and, accordingly, storage of Ca^2+^ in the ER (García-Sáez, 2012), Mfn2 can activate Bax and inhibit Bcl-2 expression. Thus, Mfn2 triggers the inhibition of the anti-apoptotic protein and the activation of the proapoptotic protein.

According to another modern concept (Dityatev and Schachner, 2003), a synapse is considered to be a four-part structure, which additionally consists of an irreplaceable component for providing neurotransmission, the extracellular matrix. However, there is no data concerning the direct connection between intracellular MERCs and the extracellular matrix related to neurotransmission.

## The Emerging Role of MERCs in Supporting Neurotransmission During the Development of Neurodegeneration

Neurotransmission becomes substantially altered during the process of neurodegeneration (Bae and Kim, 2017). Synapses and the machinery that provides the maintenance of their function are especially vulnerable to chronic stress factors observed in neurodegenerative pathologies. Taking into account the number of cellular functions carried out with MERCs, it appears to be natural that mutant forms or altered functions of several resident proteins are implicated in the course of neurological pathologies (Veeresh et al., 2019). Such pathologies include proteinopathies (Poston et al., 2013; Paillusson et al., 2016) and metabolic disorders in Wolfram syndrome (Wiley et al., 2013; Delprat et al., 2018) and GM1-gangliosidosis (Sano et al., 2009). Abnormalities in the structure of the genes encoding MERCs-localized functional protein complexes appear to be an important part of neurodegeneration. For instance, SIGMA1R and PACS2 knockdown led to the neuronal degeneration in murine hippocampal cultures, which demonstrates the important role of MERCs proteins concerning neuronal integrative properties (Hedskog et al., 2013). Therefore, MERCs proteins potentially could be used as molecular targets in creating a new neuroprotective approach. Taking that into account, the link between MERCs functions and the pathogenesis of such neurodegenerative pathologies as Alzheimer’s, Parkinson’s, and Huntington’s diseases received much attention recently due to the novel observations (De Mario et al., 2017; Moltedo et al., 2019; Magalhães Rebelo et al., 2020).

### Alzheimer’s Disease

Pathogenesis of Alzheimer’s disease (AD) serves as a good example of the emerging role of MERCs proteins in supporting synaptic transmission during the development of neurodegeneration. The loss of synapses correlates with the cognitive impairment in patients with AD and is considered to be a preceding mechanism of neuronal loss in affected brain areas (Tönnies and Trushina, 2017). In general, AD is characterized by the β-amyloid (Aβ) plaque formation and the accumulation of neurofibrillary tangles, which consist of hyperphosphorylated tau protein, in the brain. The amount of synapse-localized oligomeric Aβ correlates with excitatory synapse loss (Koffie et al., 2009). It is important to note that the oligomeric form of Aβ is considered to be the most synaptotoxic (Kayed and Lasagna-Reeves, 2013). The precursor of Aβ is the transmembrane protein APP (Amyloid precursor protein) (Maltsev et al., 2014).

The novel hypothesis of the AD pathogenesis suggests that MERCs are involved in the initial phase of the disease, and their altered functions contribute to cellular metabolic dysfunction. In support of this, MERCs are considered to be an intracellular site of γ-secretase (APP cleavage enzyme) and presenilins (γ-secretase subunit proteins) activity, which gene mutations are associated with AD pathogenesis (Area-Gomez et al., 2009; Area-Gomez and Schon, 2016). Loss of presenilins, in turn, impairs glutamatergic neurotransmission from the presynaptic side (Zhang et al., 2009).

Furthermore, it was demonstrated by Pera et al. (2017) that in addition to γ-secretase, one of the APP cleavage fragments, C99 (β-C-terminal fragment), is also localized in MAM. In line with that, the accumulation of uncleaved C99 led to physical and functional stimulation of MERCs formation, increased sphingomyelinase activity in MERCs, and, subsequently, elevated ceramide content. Ceramide, in turn, is known as a proapoptotic molecule and mitochondrial respiration inhibitor (Pera et al., 2017). The enzyme required for APP cleavage to Aβ and C99, ACAT (acyl-CoA: cholesterol acyltransferase) (Puglielli et al., 2004), is present in MAM (Area-Gomez et al., 2018). Thus, it is supposed that MERCs are the cleavage site of APP. Mutations in presenilin genes and disruption of γ-secretase activity can lead to the accumulation of C99 in MERCs, which are in direct contact with mitochondria and can affect the functional status of organelles and, indirectly, intercellular signaling via synapses (Picone et al., 2014; Schreiner et al., 2015).

The early contribution of MERCs to the progression of AD is also supported with the proteome analysis. Many MAM-localized proteins are associated with AD (Völgyi et al., 2018), though the evidence related to the change of their content and distribution in the brain appears to be controversial. The level analysis of SIGMA1R and PACS2 in mice with APP_Swe/Lon_ mutations showed that levels of both proteins are elevated in the hippocampus before Aβ plaque formation and also increased in the cerebellum and cortex, but in this case after the plaque formation (Hedskog et al., 2013). On the contrary, in postmortem cortical tissue of patients with AD the increase in PACS2 level was shown, but a decrease in the level of SIGMA1R (Hedskog et al., 2013). Besides, a life-time decline in SIGMA1R distribution was demonstrated in the cerebral cortex of patients with AD already at the early stage of the disease (Mishina et al., 2008). According to the recent evidence (Lau et al., 2020), in cortical pyramidal neurons of AD-affected brain, the complex of VAPB-PTPIP51 was shown to be disrupted, which together with previous data can outline the events leading to the altered Ca^2+^ transfer, ATP synthesis, and, subsequently, impaired function of synaptic transmission (Gómez-Suaga et al., 2019). The increase in oxidative stress reactions was observed along with the perturbed levels of MAM-localized proteins in the cortex of murine AD models (Völgyi et al., 2018), which also reflects a possible link between MERCs functions and impaired neurotransmission, since the increased production of reactive oxygen species directly affects synaptic activity in neurons (Ahmad et al., 2017).

### Parkinson’s Disease

Parkinson’s disease (PD) is a neurodegenerative disease associated with the progressive degeneration of dopaminergic neurons in the pars compacta of the substantia nigra and the formation of intracellular protein inclusions – Lewy bodies, as well as with Lewy neurites – neuronal processes characterized by dystrophy, with a significant role of the protein α-synuclein. Patients with PD suffer from motor disorders such as bradykinesia, tremors, muscle rigidity, and postural instability (Gómez-Suaga et al., 2018).

The presence of α-synuclein was observed in MAM (Guardia-Laguarta et al., 2014), while α-synuclein-lacking neurons demonstrated a decreased number of MERCs and an increased ER-mitochondria distance (Faustini et al., 2019). The evidence that mitochondrial morphology, the level of Mfn1 expression, and respiration capacity was affected in cortical neurons of mice lacking α-synuclein supports the hypothesis about a physiological interplay between this protein and the function of mitochondria (Faustini et al., 2019). In addition, oligomerization of α-synuclein induces mitochondrial toxicity, fission, energy stress, and mitophagy, demonstrating the role of α-synuclein as a therapeutic target for future studies (Ryan et al., 2015). Many observations indicate that the accumulation of α-synuclein in presynaptic terminals disrupts the functioning of synaptic proteins, synaptic plasticity, and neurotransmission, hence inducing axonal damage (Ben Gedalya et al., 2009; Cheng et al., 2011; Anichtchik et al., 2013), which can be induced directly with the effect of α-synuclein, or indirectly with MAM proteins being intermediates. On one hand, Calì et al. (2012) showed that α-synuclein supports the ER-mitochondria interaction and thus is essential for the preservation of mitochondrial Ca^2+^ homeostasis, while the mutant protein negatively influenced Ca^2+^ transfer to mitochondria. On the other hand, according to the evidence by Paillusson et al. (2017), overexpression of both wild-type and mutant α-synuclein reduced the number of ER-mitochondria connections. Moreover, the ability of mutant α-synuclein to interact with VAPB, disrupting the VAPB-PTPIP51 complex, was associated with the negative regulation of the interaction between mitochondria and the ER (Paillusson et al., 2017). Along with the previous evidence, the demonstrated ability of α-synuclein to interact with, block, and translocate mitochondrial VDAC through the outer membrane of the organelle can lead to the disruption of Ca^2+^ exchange through MAM (Rostovtseva et al., 2015). Taking into consideration that multiple α-synuclein-involving mechanisms ultimately lead to the dysregulation of MERCs-associated Ca^2+^ exchange, the expression of mutant protein in PD may strongly affect the neurotransmission in the mitochondria-associated presynapses, since mitochondrial Ca^2+^ uptake stimulates ATP production, which is crucial to satisfy local energy demands during the active synaptic transmission (Harris et al., 2012).

Mitochondria-endoplasmic reticulum contacts can fail to support the neurotransmission in PD not only due to α-synuclein mutations, but also because of the local presence of mutant proteins with impaired functions. Indeed, PD-related mutations in Miro1 (Mitochondrial Rho GTPase 1), a MERCs-localized protein (Modi et al., 2019), lead to the decreased mitochondrial transport in neurons, increased ER-mitochondrial tethering, and the adverse effect toward regulation of mitophagy and Ca^2+^ homeostasis (Berenguer-Escuder et al., 2020). Importantly, Miro1 is involved in the postsynaptic positioning of mitochondria in response to activated NMDA receptors (Macaskill et al., 2009). Concerning the significant role in Ca^2+^ exchange between the ER and mitochondria, DJ-1 protein that can induce MERCs formation was also highlighted, the dysfunction of which is associated with the development of PD. According to a recent study (Liu et al., 2019), DJ-1 appears to be an important component supporting the complex IP3R-GRP75-VDAC, providing an integrity and function of ER-mitochondria connection. Suppression of DJ-1 led to the negative regulation of Ca^2+^ exchange between organelles (Ottolini et al., 2013) and impaired the ER-mitochondria association related to interaction with the IP3R-GRP75-VDAC complex (Liu et al., 2019). Finally, as was mentioned above, SIGMA1R can bidirectionally modulate the state of NMDA receptors, thus exerting an influence on synaptic plasticity. Both experimental (Wilson et al., 2020) and theoretic (Yang et al., 2019) studies show the link between SIGMA1R and the neurotransmission in PD.

### Huntington’s Disease

Huntington’s disease (HD) is an autosomal dominant inherited neurodegenerative disorder with a manifestation at the age of 35–50 years and symptoms including motor impairment and progressive dementia. The pathogenesis of HD is associated with the accumulation of CAG repeats in exon 1 of the Huntingtin protein (Htt) gene and the formation of mutant Huntingtin (mHtt), which contains polyglutamine tract (PolyQ) at the N-terminus. During the progression of HD the accumulation of mHtt aggregates in the neuronal nucleus and, to a lesser extent, in the cytoplasm, dendrites, and axonal terminals is observed (Ghosh and Tabrizi, 2018). Even though Htt and mHtt are ubiquitously expressed in the brain, the development of HD is associated predominantly with impaired synaptic transmission (Smith-Dijak et al., 2019), selective loss of striatal neurons, and less markedly with loss of hippocampal and subthalamic neurons. At the late stage of HD the cerebral cortex is also affected (Reddy et al., 2009).

As well as in AD and PD, during the HD progression, the content of MERCs-localized proteins is changed. At the early and middle stages of HD, IP3R3, and GRP75 were shown to be reduced in the striatum, while Mfn2 was decreased only in the putamen (Cherubini et al., 2020). The supposed participation of MERCs in the pathogenesis of HD is also associated with the influence that mHtt exerts on mechanisms of ER-mitochondria Ca^2+^ exchange, Ca^2+^ capacity, and the maintenance of Ca^2+^ homeostasis in brain cells. In the cortex and striatum of murine HD models, impaired interaction between IP3R1 and GRP78 (BiP) was shown (Higo et al., 2010). wtHtt together with HAP-1 (Huntingtin-associated protein 1), or Htt separately, can form a complex with the C-terminus of IP3R1, which leads to its sensitization toward IP3 and promotes Ca^2+^ release from the ER (Tang et al., 2003). Calcium depletion in the ER, in turn, can stimulate CRAC (Calcium release-activated channels) opening and, subsequently, the store-operated calcium entry (Prakriya and Lewis, 2015), which was induced in transgenic HD mice (Vigont et al., 2014, 2015). Htt and mHtt localization in striatal cells were associated with the outer mitochondrial membrane (Choo et al., 2004). Accordingly, Ca^2+^ capacity, the amount of Ca^2+^, which mitochondria can store before mitochondrial permeability transition pore opening and the release of proapoptotic factors into the cytoplasm, was significantly reduced after the PolyQ (Panov et al., 2002) and truncated N-terminal mHtt (Choo et al., 2004) exposure. Finally, under conditions of impaired Ca^2+^ homeostasis, the pathological fragmentation of mitochondria is observed (Shirendeb et al., 2012) upon interaction between mHtt and Drp1 (Costa et al., 2010). The evidence provided by Cherubini et al. (2020) supports the hypothesis that increased mitochondrial fragmentation and reduced ER-mitochondria association in HD striatum are interconnected. Moreover, a possible connection between impaired mitochondrial dynamics, reduced synaptic activity, and the proteins of MERCs in HD was demonstrated (Manczak and Reddy, 2015). Interestingly, the treatment with Mdivi1 (mitochondrial division inhibitor 1) rescued abnormally up-regulated genes of MERCs-related fission proteins (Drp1, Fis1), down-regulated fusion genes (Mfn1, Mfn2), which not only subsequently normalized the function of mitochondria but also led to the up-regulation of synaptic genes in mHtt-neurons (Manczak and Reddy, 2015). Thus, according to the findings mentioned above, the impaired function of several MERCs proteins can contribute to the mitochondrial dysfunction and neuronal vulnerability and loss observed in HD. However, the data presented up to date are limited; hence the role of MERCs in the contribution to altered neurotransmission in HD requires further study.

## Discussion

In the last few years, multiple studies of mitochondria-endoplasmic reticulum interactions in neuronal cells were carried out, and valuable data that elucidate the role and functions of MERCs related to synaptic transmission were obtained (Gómez-Suaga et al., 2019; Dematteis et al., 2020; Liao et al., 2020; Perrone et al., 2020). Numerous data confirm the participation of MERCs in the regulation of cell functions such as calcium and lipid homeostasis, fission and mobility of mitochondria, autophagy, and the initiation of apoptosis, in particular, in neuronal cells. The data altogether reflect the potential implication of MERCs in supporting active neurotransmission, indicating the role of these intracellular contacts as an assistant to neurotransmission. Even though MERCs can be present inside the vast majority of neuronal cells and in different parts of the synapse, their role in the neurotransmission may be underestimated or not taken into account.

The analysis of literature related to the topic revealed several issues connected with studying MERCs in neuron-glial networks. The data are insufficient not only to separate the function of proteins in intracellular contacts and free membranes, but also to characterize the differences of MERCs in various parts of the neuron, types of glial cells, and brain areas. There is still a lack of elegant experiments elucidating the role of MERCs precisely in synaptic transmission. Despite multiple studies carried out, there are still several questions that have yet to be answered. For example, is there but one type of MERCs that could be functionally modified, or MERCs with different cleft width fulfill specific functions from the very beginning without the possibility of restructuring? It also appears to be yet unknown, whether some of the described proteins are localized directly in MAM or if their presence in the MAM’s fraction is merely a consequence of the imperfect fractionation methods. New methods, such as split-BioID (Kwak et al., 2020) that can detect MAM’s regulatory proteins and reveal the proteome of synaptic intracellular contacts are required. Finally, it remains to be poorly understood whether altered MERCs functions are primary or secondary to the development of neurodegeneration. The MERC’s role of assistant to neurotransmission confirmed, but still requires further study.

## Author Contributions

OS designed the study. OS and PP collected and analyzed the data, wrote and revised the manuscript. IM supervised the study and conceptualized the idea. All authors contributed to the article and approved the submitted version.

## Conflict of Interest

The authors declare that the research was conducted in the absence of any commercial or financial relationships that could be construed as a potential conflict of interest.
